# Healing potentiality of blood clot, S-PRF and A-PRF as scaffold in treatment of non-vital mature single rooted teeth with chronic peri-apical periodontitis following regenerative endodontic therapy: randomized clinical trial

**DOI:** 10.1186/s12903-024-05378-0

**Published:** 2025-01-09

**Authors:** Omnia Badawy Darwish, Said Mohamed Abdel Aziz, Hany Samy Sadek

**Affiliations:** 1 Endodontics Department, Faculty of Dentistry, Cairo University, 11 El-Saraya Street, Manial, Cairo, Egypt; 2 Endodontics Department, Faculty of Dentistry, Menofia University, Shibin Al kawm, Menofia, Egypt; 3 Endodontics Department, Faculty of Dentistry, Cairo University, Cairo, Egypt

**Keywords:** Necrotic teeth, Mature teeth, Regeneration, Revascularization, Regaining sensibility

## Abstract

**Objectives:**

This randomized prospective controlled trial investigated the effectiveness of different strategies of regenerative endodontic therapy on necrotic mature anterior teeth with chronic periapical periodontitis with 18 months follow up.

**Methods:**

A total analyzed 51 adult participant with mature single rooted teeth having necrotic pulp with chronic periapical periodontitis (PAI ≥ 3) were selected. Patients had been randomly categorized into three distinct groups (*n* = 17 each group). All groups received the same treatment on the first visit. After 2 weeks, regenerative treatment was performed using either blood clot technique, Standard-PRF and Advanced-PRF approach. A standardized radiograph was taken, and the patients instructed for 6, 12 and 18 months follow up periods. Fisher's exact test was applied to compare the frequency of PAI scores at different follow-up intervals between the three groups.

**Results:**

The results showed radiographic success at 18 months (58.8% in blood group, 94.1% in S-PRF group and 76.5% in A-PRF group). There was no statistically significant difference between the three groups according to incidence of healing at 6, 12 and 18 months. Clinical success was 82.4% in blood group and 88.2% in both S-PRF and A-PRF groups. There was no significant difference between the three groups (*p* = 1). The overall success (clinical and radigraphic) was 76.5%. Incidence of the gaining sensitivity after 12 and 18 months was 29.4% with A-PRF group and 41.2% within the S-PRF group, 17.6% with BC group.

**Conclusion:**

PRF based regenerative technique may outperform the blood clot technique in treatment of non-vital mature teeth with chronic periapical periodontitis. There is a need for future randomized clinical studies to consolidate procedures in this field with more prolonged evaluation periods.

**Clinical trial registration:**

The study was retrospectively registered with ClinicalTrials.gov (ID: NCT04606719) in 28/10/2020.

## Introduction

Periapical periodontitis is an inflammatory disease occurs as a result of infection of the tooth pulp tissue following carious lesions or traumatic injuries [1]. The traditional treatment of this condition is apexification for immature teeth and conventional root canal treatment for mature teeth.

The goal of an apexification in immature teeth is to encourage the development of an apical barrier with calcium hydroxide or obturate with calcium silicate material [2, 3]. However, the constraints of apexification are that it can’t promote maturation of root apex,and can’t increase the thickness of the dentinal walls to strengthen the fragile immature tooth [4].

Regrading conventional root canal treatment for mature teeth, the teeth become brittle after endodontic treatment,as there is a tendency for development of dental cracks which often associated with worse prognosis and extraction is typically necessary in these cases. Additionally, improper biomechanical preparation increases the chance of periapical reinfection. These drawbacks highlight how important pulp tissue is to maintaining dental health. Vital pulp tissue contributes to the tooth's tensile strength, prevents infection, and helps produce reparative dentin [5].

A promising new alternative for apexification and conventional endodontic treatments is regeneration therapy. Östby pioneered the idea of inducing blood into root canals to rejuvenate dental pulp tissue in 1961 [6]. American Association of Endodontists (AAE) expressed the regenerative endodontic procedures (REPs) as “biologically based procedures designed to replace damaged structures, including dentin and root structures, as well as cells of the pulp-dentin complex”[7].

Regenerative endodontic techniques are particularly effective for treating immature permanent teeth with necrotic pulps but there was no significant difference concerning clinical and radiographic outcomes and survival rate outcomes between revascularization and apexification [8].

However subsequently this, several studies have been carried out suggesting that REPs may eventually become an effective therapy option for mature teeth with pulp necrosis and apical periodontitis [9–13].

While REPs conforms to the general principles of conventional root canal therapy, there are some subtle modifications, such as a disinfection procedure that only uses chemical irrigation and takes into account the potential cytotoxicity of the cells being recruited for the canal and stimulates pulp-dentin regeneration by having an abundance of scaffolding materials that have the ability to migrate, proliferate, and display the spatially distributed group of cells necessary for the substitution of the target tissue's structure as well as its function [14].

The blood clot performs as a scaffold, and the growth factors within it attract stem cells, occasionally from the periapical papilla. Unavoidably, erythrocytes found in the clot of the blood column endure necrosis, altering its properties [15]. To overcome the disadvantages associated with the blood clot revascularization technique, autologous platelet concentrates (APCs) have lately gained attention as a potential technique for promoting regeneration. Platelet Rich fibrin(PRF), a second-generation platelet concentrates was developed by Choukroun et al. 2001 [16]. Latest in vitro investigations have shown that PRF enhances the migration, proliferation and differentiation of stem cells from the apical papilla (SCAPs) [17, 18].

With the ultimate goal of increasing platelet, leucocyte counts and growth factors, Ghanaati et al. 2014 proposed a low-speed centrifugation theory that resulted in an improved PRF called Advanced PRF [19]. Furthermore, Kobayashi et al. [20] determined that A-PRF provided a maximum quantity of growth factors throughout an extended time frame from 15 min to 10 days when compared to PRF thereby seems to be valuable for regenerative procedures.

To our knowledge, just one study comparing blood clot and standard PRF found that both groups' PAI scores of periapical lesions decreased significantly in the first 6 and 12 months after a REP. At 6 and 12 months, S-PRF showed a better periapical lesion healing rate than BC [11].

Thus, the goal of this randomly allocated controlled trial is to investigate and distinguish between the healing potentiality and regaining sensitivity of rejuvenating endodontic procedures for devitalized mature single-rooted teeth with chronic periapical periodontitis using a variety of scaffolds. The null hypothesis of this randomized clinical trial was that there would be no difference in periapical healing and regianing sensitivity outcomes between the blood clot, standard PRF, and Advanced PRF as scaffolds in treatment of non-vital mature single rooted teeth with chronic periapical periodontitis following regenerative endodontic therapy.

## Subjects & methods

### Study design & ethical approval

This randomized clinical study followed the CONSORT guidelines (http://www.consort-statement.org/) for reporting clinical trials in Endodontics. This clinical trial was retrospectively registered on https://clinicaltrials.gov/study/NCT04606719 in 28/10/2020. The Cairo University faculty of dentistry's institutional review board gave acceptance for this study (Approval number: REC reference: 1–12-20). Written informed consent was obtained from all study participants after the study methodology was explained.

### Sample size calculation

The sample size was estimated assuming 80% study power and 5% alpha error. Based on Shivashankar et al.2017 [21], the mean (sd) Orstavik PAI healing score of the blood column group was 2 (0.6). Aiming to detect a minimum clinically significant difference of 1 score, the required sample size was calculated to be 7 experimental participants and increased to 14 to make up for any drop out. The sample size was calculated using G*Power (Version 3.9.1.7).

### Eligibility criteria

The eligibility criteria for the present study were Medically free patients with age between 18–35 years old with no sex predilection. Patients with asympotomatic necrotic pulp in mandibular & maxillary anterior single rooted permanent teeth with mature apex that has PAI ≥ 3 were included.

Patients with systemic diseases, pregnant women, patients that have allergy to ciprofloxacin or metronidazole and teeth with radiographic signs of severe root resorption, or root fracture or associated with periodontal disease were excluded form the study.

### Randomization

Computer software was used to create a simple random sequence (http://www.random.org/).The colleague (H.S) carried the randomization table, which the operator (O.D) applied to determine the patient's group assignment only at the time of grouping (at the subsequent visit). Both the operator and the patients cannot be blinded due to the nature of the interventions as the control group depends on initiating bleeding while the intervention groups need withdrawal of blood samples from the patients. The allocation concealment method was done as the participants dragged opaque sealed envelopes with a random number inside that indicated which regenerative treatment would be used to them at the start of the second visit.

### Patient pre-operative examination

Patients were recruited from the outpatient clinic of the Department of Endodontics, Faculty of Dentistry, Cairo University, Cairo, Egypt from Novmber 2020 until December 2022. The operator (O. D) collected medical and dental history with clinical examination data from all patients in this study. Tentative clinical examination for the suspected tooth using a diagnostic mirror and probe for assurance of presence of caries or old restoration or fractured crown also percussion test was performed. Then, radiographic examination was done using intra-oral bisecting angle technique with a periapical digital sensor plate to assure root morphology as the absence of root curvature, calcifications and resorption and the presence of periapical radiolucency. Consequently, the diagnosis of necrotic single rooted teeth with periapical radiolucency was confirmed clinically by negative response to an electrical pulp tester and cold test. When the participant was diagnosed as necrotic pulp with chronic apical periodontitis after tentative clinical and radiographic examination. The patients who were admitted into the experiment and approved by signing the consent form, were given a description of regenerative procedure which took place over two visits.

### Regenerative endodontic procedures

**Fig. 1:**
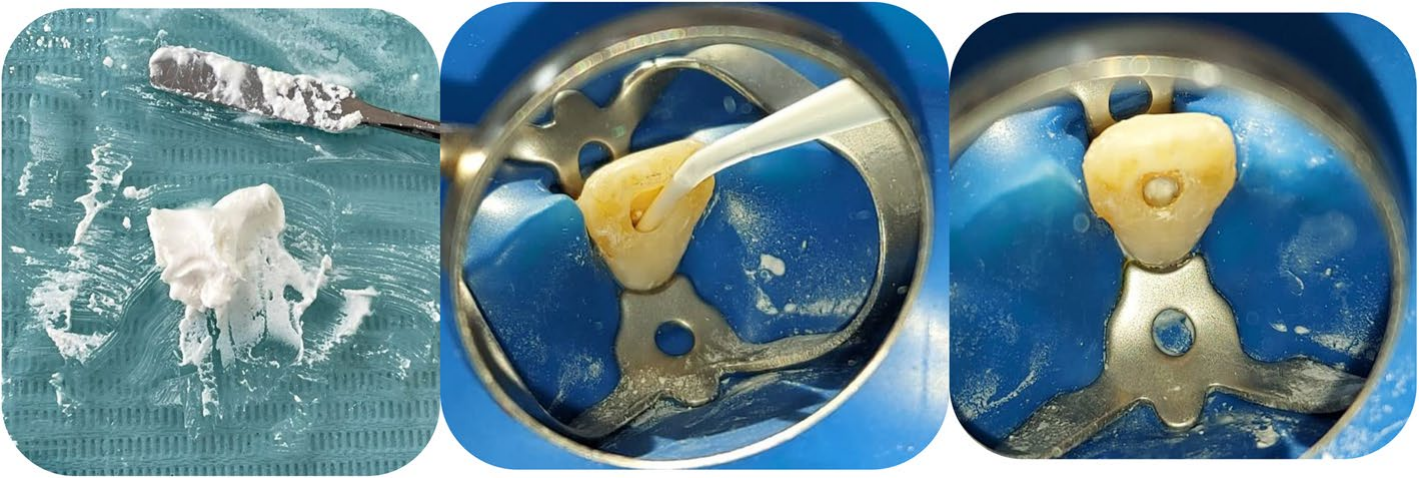
Double antibiotic paste (DAP) was prepared by grinding one tablet of metronidazole (500 mg) and one tablet of ciprofloxacin (500 mg), which was mixed with saline to form a homogenous paste with creamy consistency. The mix injected into the canal just below the cemento-enamel junction (CEJ)

*At the first visit*; local infiltration anesthesia of 1.8 ml of 2% Mepivacaine HCl with 1:100,000 epinephrine *(Alexandria Company for Pharmaceuticals and Chemical Industries, Egypt)* was used. Rubber dam was used to isolate the tooth, and the access cavity preparation was done. Stainless steel hand K-files in sizes # 10 and #15 *(Mani, Inc., Utsunomiya, Tochigi, Japan)* were used for canal negotiation and patency inspections. Using an electronic apex locator *(Root ZX, J. Morita, Irvine, California, USA)* to determine the working length, which was then verified to be 0.5–1 mm shorter than the radiographic apex by intraoral periapical radiography. In accordance with the manufacturer's instructions, ProTaper Next rotary instruments *(Dentsply Maillefer, Maillefer, USA)* were used in an endodontic motor to mechanically prepare root canal (X3 for mandibular anterior teeth and X4 for maxillary anterior teeth) utilizing the crown-down approach. The canals were irrigated with 2.6% sodium hypochlorite *(Dent House, Medical company, Cairo,Egypt)* using a 30-gauge single side vented needle *(Zogear Products, Shanghai, China)* placed approximately 1 mm from the root end. This was done in between two consecutive files. After saline irrigation, paper points were used to dry the canal. Double antibiotic paste was prepared by grinding one tablet of metronidazole (500 mg) and one tablet of ciprofloxacin (500 mg) in a ratio 1:1, which was mixed with saline to form a homogenous paste with creamy consistency [22]. The mix was injected into the canal approximately 1 mm from the root end till just below the cemento-enamel junction (Fig. 1). A sterile cotton pellet was placed into the pulp chamber then the access cavity was sealed by glass ionomer cement. The second appointment was scheduled after resolving of any signs and symptoms after 2–3 weeks.

*At the second visit*, A plain anesthesia 1.8 ml 3% mepivacaine was administered to anaesthetize the relevant nerve. Prior to removing the glass ionomer with a high-speed handpiece, the rubber dam was positioned. Under dental operating microscope, the double antibiotic paste was removed by copious irrigation with 20 ml of 2.6% sodium hypochlorite followed by 20 ml of 17% EDTA *(MD-Cleanser™, Metabiomed. C.O. Korea)* separated with saline then the canals were dried using paper points. The patients were randomly divided into 3 groups:

#### The control group (blood clot)

Following the canals being dry, pre-curved K-file # 25 or # 30 was intentionally used to over-instrumentation 2–3 mm beyond the apex into the periapical region, causing bleeding adjacent to the apical foramen to be approximately below CEJ. The participant was eliminated from the research if the bleeding induction failed. The semi-coagulated blood clot was covered by a collagen sponge placed inside the canal to prevent the bioactive material from being placed apically. A 3-mm-thick layer of white tri-calcium silicate biomaterial (Bio-C®) *(Angelus Indústria de Produtos Odontológicos, Brazil)* was placed over the collagen sponge, and it was gently adapted to dentinal walls using moistened cotton.

#### The intervention group I (S-PRF)

**Fig. 2:**
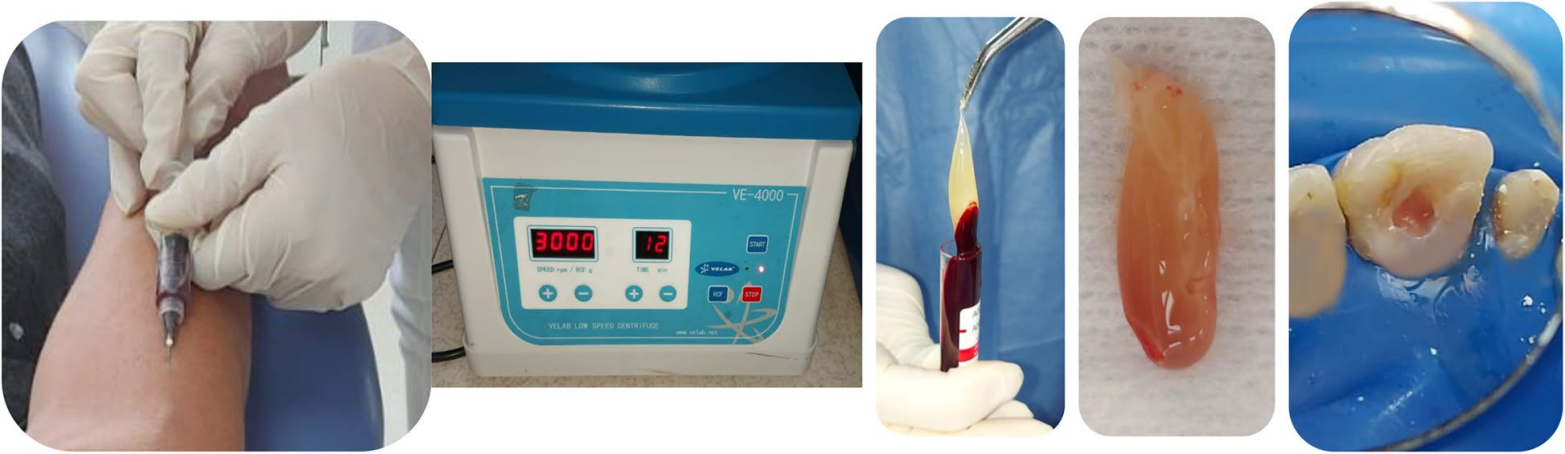
5 mL of venous blood was taken from the participant in dried glass test tube and immediately centrifuging it at 3000 rpm for 12 min. Application of S-PRF inside the tooth

Standard platelet-rich fibrin was generated by taking 5 mL of venous blood from the patient into a drying glass container and by spinning it at 3000 rpm for a duration of ten minutes (Fig. 2). Upon centrifugation, three different layers were formed in the test tube: the bottom layer of RBCs, the topmost layer of a-cellular plasma, and a platelet-rich fibrin clot in the center. S-PRF was introduced into the canal using pluggers till reach beyond CEJ then collagen sponge was inserted and 3-mm-thick layer of white tri-calcium silicate biomaterial (Bio-C®) *(Angelus Indústria de Produtos Odontológicos, Brazil)* was applied.

#### The intervention group II (A- PRF)

**Fig. 3:**
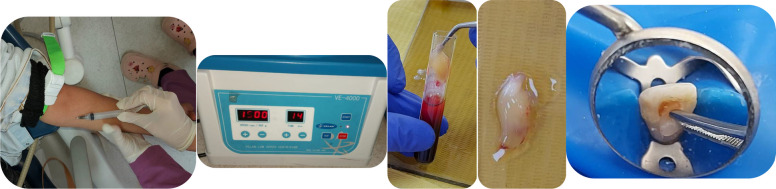
5 mL of venous blood was taken from the participant in dried glass test tube and immediately centrifuging it at 1500 rpm for 14 min. Application of A-PRF inside the tooth

The newly generated A-PRF involves extracting 5 mL of venous blood from the patient into a dried glass test tube instantly being spun at 1500 rpm for 14 min (Fig. 3). A-PRF was applied into the canals till reach beyond CEJ then collagen sponge was inserted and 3-mm-thick layer of white tri-calcium silicate biomaterial (Bio-C®)*(Angelus Indústria de Produtos Odontológicos, Brazil)* was placed.

For all cases, glass-ionomer cement was placed over the cervical barrier then the tooth was restored using resin composite restoration. Employing a specially designed index made up of a silicon impression material *(Aquasil, Dentsply Sirona),* a paralleling device *(Dentsply Sirona, Milford, DE, USA),* and a Rinn film holder; postoperative intra oral periapical radiograph was taken as baseline record and the patient was given a follow-up appointment every six months and informed of the significance of the long-term follow-up assessment.

### Assessment of treatment outcomes

All patients were recalled at 6, 12 and 18 months. Their treated teeth were evaluated clinically and radiographically. Absence of pain and swelling, disappearance of sinus tract and no evidence of soft tissue destruction are the criteria of the *clinical success*.

*The primary outcome* was radiographic healing which assessed using periapical index score that provide an ordinal scale of 5 scores as follows [23]:

1. Normal periapical anatomy.
2. Mild changes in bone pattern.
3. Changes in bone pattern with diffuse loss of mineral.
4. Apical Periodontitis with definite radiolucency in the periapical area.
5. Severe periodontitis with features of exacerbation.

Follow-up radiographs of each case were evaluated by two independent observers who were blinded from the groups. The calibration results of the two examiners' calibrations were compared, and re-evaluation was performed in case of disagreement until an inter-agreement was established.

Restoring pulp sensitivity was the *secondary outcome* of examining electric pulp tester responses (yes or no).

### Statistical analysis

The Shapiro Wilk test was used to check the normality of the data. The mean, standard deviation (SD), median, minimum (min), and maximum (max) values were used to display continuous data. When comparing non-normally distributed data between groups, the Kruskal Wallis test was employed. Fisher exact tests and Chi square tests were used to analyze the frequencies (N) and percentages (%) of the categorical data. A significant level of 0.05 was used to each test. With SPSS software, statistical analysis was carried out (IBM Corp. Released 2017. IBM SPSS Statistics for Windows, Version 25.0. Armonk, NY: IBM Corp.)

## Results

**Fig. 4:**
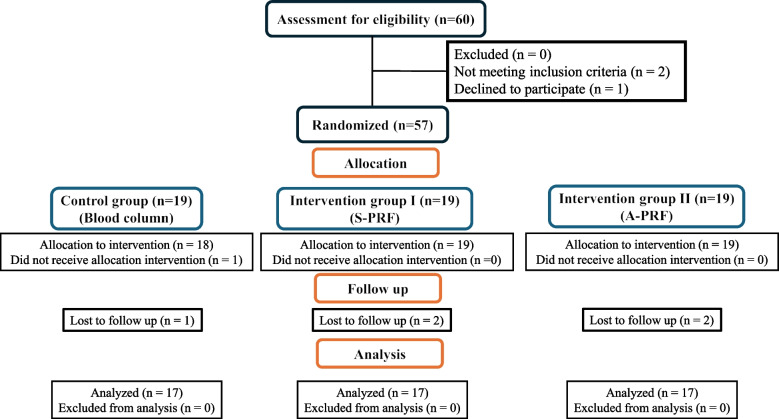
CONSORT 2010 flow diagram of the study

Sixty participants were selected for the study from the endodontic department's outpatient clinic after being evaluated to figure out if they met the requirements. Fifty-seven patients who met the eligibility criteria were selected for the. Nineteen patients were randomly assigned to each of the three groups (blood column, Standard PRF, and Advanced PRF). Figure 4 displays the study's CONSORT flow diagram. Two patients were excluded as one of them refused to participate due to the long follow-up period and the second, after allocated to control group, couldn’t initiate bleeding from periapical area.

Table 1 summarizes the demographic and baseline clinical and radiographic characteristics of 17 enrolled patients including age, gender, tooth location, etiology of pulp necrosis, number of teeth involved in the lesion and baseline periapical index scores.

**Table 1:** The demographic and baseline clinical and radiographic characteristics. Mean, standard Deviation (SD), frequencies (n), percentages(%), results of Kruskal Wallis test for comparison of age, results of Chi square test for comparison of gender distribution, tooth location distribution and etiology of pulp necrosis and results of Fisher’s exact test for comparison of number of teeth involved and incidence of baseline PAI scores between the three groups

Baseline data	A-PRF (*n* = 17)	S-PRF (*n* = 17)	BC (*n* = 17)	*p-*value*
**Age (Years)**				
*Mean (SD)*	26.06 (6.16)	24.76 (6.53)	23.24 (6.9)	0.185
**Gender [n(%)]**				
Males	1 (5.9%)	6 (35.3%)	8 (47.1%)	0.025*
Females	16 (94.1%)	11 (64.7%)	9 (52.9%)	
**Tooth location distribution[n(%)]**				
Maxilla	*11 (64.7%)*	*11 (64.7%)*	*11 (64.7%)*	1.0
Mandible	6 (35.3%)	6 (35.3%)	6 (35.3%)	
**Etiology of necrosis[n(%)]**				
Caries	5 (29.4%)	6 (35.3%)	6 (35.3%)	0.916
Trauma	12 (70.6%)	11 (64.7%)	11 (64.7%)	
**Number of teeth involved [n(%)]**				
1 tooth	15 (88.2%)	13 (76.5%)	15 (88.2%)	0.704
2 teeth	2 (11.8%)	4 (23.5%)	2 (11.8%)	
**Baseline PAI scores [n(%)]**				
Score 3	2 (11.8%)	2 (11.8%)	0 (0.0%)	0.435
Score 4	10 (58.8%)	7 (41.2%)	8 (47.1%)	
Score 5	5 (29.4%)	8 (47.1%)	9 (52.9%)	

^*^P value indicate significance (*P* ≤ 0.05)

**Fig. 5:**
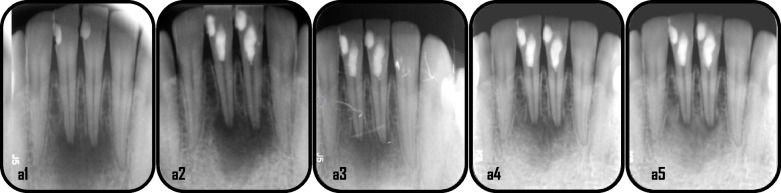
Representative cases of blood column group revealed healing process for mandibular right & left central incisors: a1: preoperative x-ray (PAI = 5), a2: immediate postoperative, a3: 6 months follow-up(PAI = 4), a4: 12 months follow up (PAI = 3), a5: 18 months follow up (PAI = 2)

**Fig. 6:**
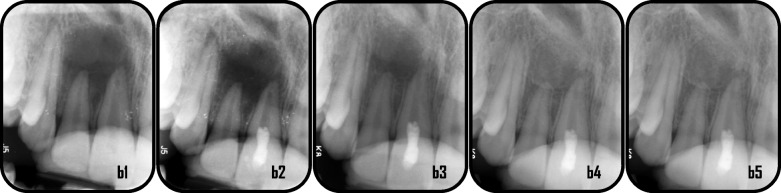
Representative case of blood column group revealed healing process for maxillary right central incisor: b1: preoperative x-ray(PAI = 5), b2: immediate postoperative, b3: 6 months follow-up(PAI = 4), b4: 12 months follow up(PAI = 2), b5: 18 months follow up (PAI = 2)

**Fig. 7:**
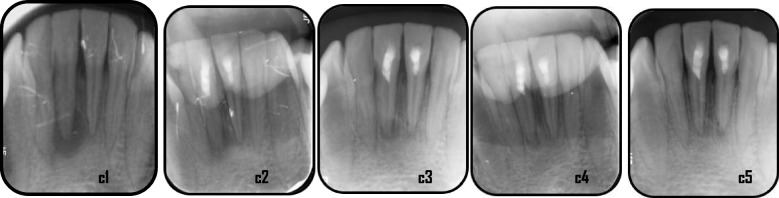
Representative case of Standard PRF group revealed healing process for mandibular right & left central incisors: c1: preoperative x-ray (PAI = 5), c2: immediate postoperative, c3: 6 months follow-up (PAI = 4), c4: 12 months follow up (PAI = 2), c5: 18 months follow up (PAI = 2)

**Fig. 8:**
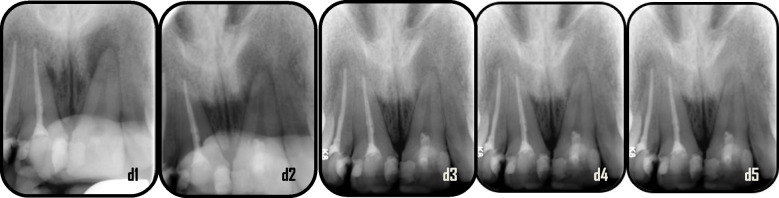
Representative case of Standard PRF group revealed healing process for maxillary left central incisors: d1: preoperative x-ray (PAI = 5), d2: immediate postoperative, d3: 6 months follow-up (PAI = 4), d4: 12 months follow up (PAI = 2), d5: 18 months follow up (PAI = 1)

**Fig. 9:**
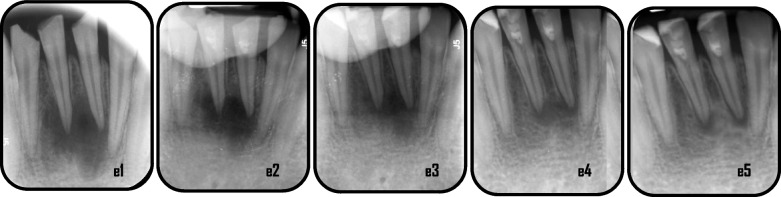
Representative case of Advanced PRF group revealed healing process for mandibular right & left central incisors:e1: preoperative x-ray (PAI = 5), e2: immediate postoperative, e3: 6 months follow-up (PAI = 4), e4: 12 months follow up (PAI = 3), e5: 18 months follow up (PAI = 3)

**Fig. 10:**
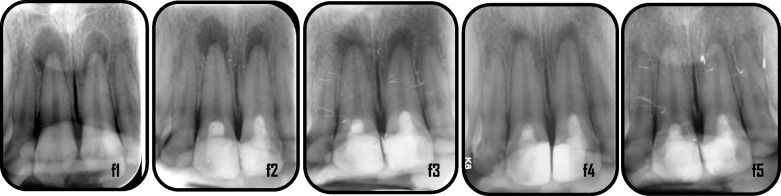
Representative case of Advanced PRF group revealed healing process for maxillary right & left central incisors: f1: preoperative x-ray(PAI = 4), f2: immediate postoperative, f3: 6 months follow-up (PAI = 4), f4: 12 months follow up (PAI = 3), f5: 18 months follow up (PAI = 2)

Representative cases of enhanced periapical healing in blood column group are shown in (Figs. 5 and 6), for Standard PRF group (Figs. 7 and 8) and for Advanced PRF group (Figs. 9 and 10). The results of Fisher's exact test for comparison of PAI scores at 6, 12, and 18 months follow up between the three groups, which revealed there was no significant difference between them (Table 2). At 18 months folllow-up, within the A-PRF group, 5 out of 17 patients (29.4%) had score 1, 8 patients (47.1%) had score 2, 2 patients (11.8%) had score 3 and 2 patients (11.8%) had score 5, while within the S-PRF group, 7 out of 17 patients (41.2%) had score 1, 9 patients (52.9%) had score 2, and 1 patient (5.9%) had score 5, and within the BC group, 2 out of 17 patients (11.8%) had score 1, 8 patients (47.1%) had score 2, 5 patients (29.4%) had score 3, 1 patient (5.9%) had score 4, and 1 patient (5.9%) had score 5. There was no significant difference between the three groups (*p* = 0.173).

**Table 2:** Frequencies (n), percentages(%) and results of Fisher’s exact test for comparison of incidence of PAI scores at 6, 12 and 18 months follow up between the three groups

	A-PRF (*n* = 17)		S-PRF (*n* = 17)	BC (*n* = 17)	*p-*value*
PAI scores at 6 months follow-up [n(%)]					
**Score 1**	0 ( 0.0%)		1(5.9%)	0 (0.0%)	0.442
**Score 2**	2 (11.8%)		0 (0.0%)	0 (0.0%)	
**Score 3**	5 (29.4%)		6 (35.3%)	4 (23.5%)	
**Score 4**	10 (58.8%)		10 (58.8%)	13 (76.5%)	
PAI scores at 12 months follow up [n(%)]					
**Score 1**		3 (17.6%)	1 (5.9%)	1 (5.9%)	0.204
**Score 2**		4 (23.5%)	10 (58.8%)	5 (29.4%)	
** Score 3**		8 (47.1%)	5 (29.4%)	10 (58.8%)	
**Score 4**		0 (0.0%)	0 (0.0%)	1 (5.9%)	
**Score 5**		2 (11.8%)	1 (5.9%)	0 (0.0%)	
PAI scores at 18 months follow up [n(%)]					
**Score 1**	5 (29.4%)		7 (41.2%)	2 (11.8%)	0.173
**Score 2**	8 (47.1%)		9(52.9%)	8 (47.1%)	
**Score 3**	2 (11.8%)		0 (0.0%)	5 (29.4%)	
**Score 4**	0 (0.0%)		0 (0.0%)	1 (5.9%)	
**Score 5**	2 (11.8%)		1 (5.9%)	1 (5.9%)	

^*^P value indicate significance (*P* < 0.05)

According to Rajasekhar et al., [24] periapical index score has been dichotomized as “success/healthy” for scores 1 and 2 and “diseased/failure” for scores ranging from 3–5. Therfore, the results of Fisher’s exact test for comparison of incidence of overall success (PAI score < 3) and failure (PAI score ≥ 3) at 6,12 and 18 months follow up between the three groups are represented in (Table 3).

**Table 3:** Frequencies (n), percentages(%), and the results of Fisher’s exact test for comparison of incidence of overall success (PAI score < 3) and failure (PAI score ≥ 3) at 6,12 and 18 months follow up between the three groups

	**A-PRF** (***n*=17)**	**S-PRF** (***n*=17)**	**BC** **(*n*=17)**	*p-*value*
**PAI scores at 6 months follow-up [n(%)]**				
**PAI score <3 **	2 ( 11.8 % )	1(5.9%)	0 (0.0%)	0.764
**PAI score ≥3**	15 (88.2 %)	16 (94.1%)	17 (100.0%)	
**PAI scores at 12 months follow up [n(%)]**				
**PAI score <3**	7 (41.2%%)	11 (64.7%)	6 (35.3%)	0.292
**PAI score ≥3**	10 (58.8%)	6 (35.3%)	11 (64.7%)	
**PAI scores at 18 months follow up [n(%)]**				
**PAI score <3**	13 (76.5%)	16 (94.1%)	10 (58.8%)	0.061
**PAI score ≥3**	4 (23.5%)	1(5.9%)	7 (41.2%)	

*P value indicate significance (*P*<0.05)

The secondary outcomes are shown in Table 4 which include clinical success as within the A-PRF group, 15 out of 17 patients (88.2%) showed clinical success and 2 patients (11.8%) did not as cervical fracture was occurred, while within the S-PRF group, 15 out of 17 patients (88.2%) showed clinical success and 2 patients (11.8%) did not as intra-oral fistula was developed, and within the BC group, 14 out of 17 patients (82.4%) showed clinical success and 3 patients (17.6%) did not as 2 of them developed swelling and fenestration was occurred with the third one. There was no significant difference between the three groups (*p* = 1).

**Table 4:** The secondary Outcomes: frequencies (n), percentages(%),results of Fisher’s exact test for comparison of clinical success and results of Chi square test for comparison of incidence of pulp sensitivity at 6,12 and 18 months follow up between the three groups

	A-PRF (*n* = 17)	S-PRF (*n* = 17)	BC (*n* = 17)	*p-*value*
Clinical success [n(%)]				
Yes	15 (88.2%)	15 (88.2%)	14 (82.4%)	1.0
No	2 (11.8%)	2 (11.8%)	3 (17.6%)	
Incidence of pulp sensitivity at 6 months [n(%)]				
Yes	0 (0.0%)	1 (5.9%)	0 (0.0%)	1.0
No	17 (100.0%)	16 (94.1%)	17 (100.0%)	
Incidence of pulp sensitivity at 12 months [n(%)]				
Yes	5 (29.4%)	7 (41.2%)	3 (17.6%)	0.322
No	12 (70.6%)	10 (58.8%)	14 (82.4%)	
Incidence of pulp sensitivity at 18 months [n(%)]				
Yes	5 (29.4%)	7 (41.2%)	3 (17.6%)	0.322
No	12 (70.6%)	10 (58.8%)	14 (82.4%)	

^*^P value indicate significance (*P* < 0.05)

Also, incidence of pulp sensibility regaining by evaluating the answers to the electric pulp tester (yes or no) at different follow-up periods are represented in Table 4. At both 12 and 18 months intervals, within the A-PRF group, 5 out of 17 patients (29.4%) showed pulp sensitivity and 12 patients (70.6%) did not, while within the S-PRF group, 7 out of 17 patients (41.2%) showed pulp sensitivity and 10 patients (58.8%) did not, and within the BC group, 3 out of 17 patients (17.6%) showed pulp sensitivity and 14 patients (82.4%) did not. There was no significant difference between the three groups (*p* = 0.322).

## Discussion

Regenerative endodontic therapy, which replaces damaged tissue instead of restoring it, has been called a "turning point" in treating necrotic teeth with periapical periodontitis. Regenerated dental pulp, unlike endodontically treated teeth, restores immune cells to the root canal's innate immunity, reducing the risk of reinfections, hydrating teeth, and strengthening their mechanical resistance to fractures [5].

According to a recent umbrella review, REPs present a promising substitute for conventional apexification techniques for immature teeth, with paletlet concentrate showing better results in periapical healing than traditional blood clots [25]. Additionally, REPs seem to be a good therapeutic option for adult necrotic teeth with periapical lesions at this stage, according to moderate quality investigation [26]**.** However, the inconsistent results highlight the necessity of standardized procedures and consistent outcome measures to more accurately assess the effectiveness of various scaffolds in RET.

Therefore, the purpose of this study was to use a randomized clinical trial to treat 51 patients with necrotic pulp and apical periodontitis in mature single-canal teeth. The three different scaffolds used in regenerative endodontic procedures; blood clot, standard PRF, and advanced PRF strategy were evaluated in this study to assess their radiographic and clinical outcomes. Our study results showed that mature teeth with a necrotic pulp and apical periodontitis might gain advantage from treatment with REPs applying any of the three strategies.

To standardize this approach, only single anterior mature tooth was used, as in most clinical studies on adult tooth REP [9–11]. Patients' ages were chosen to range from 18 to 35, as physiological alterations occur with ageing as secondary dentin and cementum deposition increases in the apical region of the tooth, resulting in apical foramen constriction, hyper-cementosis, and periodontal ligament stenosis and fibrosis. As a result, these physiological alterations may inhibit the migration of resident stem cells from the surrounding tissue into the root canal. In fact, the ageing process produces physical malfunctions of many cellular and molecular events that result in a loss of immunity and increased inflammation in the aged tissues and surrounding niche, which may hinder the regenerative process and reduce stem cell function [27, 28]. Although, al Arslan et al. observed no effect of age on regeneration outcomes in a regressive investigation of confounding characteristics [9]. Also, Gender was not a confounding factor in this study because it did not affect mesenchymal stem cells' gene expression indicators [29].

Regenerative endodontics can be successful when the root canal system's pathogenic organism and byproducts are eradicated using chemo-mechanical preparation. Like previous studies [9–11, 30, 31], the current study used full mechanical instrumentation during the first visit to remove contaminated tissue and make space for irrigants, which decontaminate, detoxify bacterial antigens, eliminate intracanal medicaments, and condition the root surface for scaffolding. The current study used 2.6% sodium hypochlorite since 6% showed cytotoxic effects on dentin, which is essential to healing [32]. The release of embedded growth factors from 17% EDTA boosts stem cell motility, angiogenesis, proliferation, and differentiation into odontoblast-like cells [33, 34]. Double antibiotic paste (DAP) was used for further disinfection, DAP had better tissue development, vascularity, inflammation control, and minimal tooth discolouration than TAP [30]. However, a recent meta-analysis of the regeneration treatment's clinical and radiological success in permanent adult teeth found that all studies had similar success rates, regardless of final irrigants or intracanal medication [35].

Blood columns (BCs) are the most popular scaffold but producing the correct blood clot is not always attainable, which could inhibit stem cells from migrating. BC’s brittle mechanical construction may not be enough to fill the root canal after treatment, causing coronal sealing collapse[34]. The cell-free technique proposes using autologous platelet products like standard or Advanced PRF as scaffolds in the root canal to enclose blood components and platelets as signaling molecules and allow cells to generate vital tissue [13, 36].

Standardized and calibrated 2-dimensional radiographic examination was done for REPs evaluation [37]. Further,the apical lesions were assessed using periapical index scoring(PAI), since PAI scoring offers a reliable and precise measuring method [38]. According to the current investigation, the radiographic healing of the periapical lesion appears encouraging. At 12 months, it showed radiographic periapical healing success (PAI < 3) (35.3% in the blood group, 64.7% in the S-PRF group, and 41.2% in the A-PRF group). At 18 months, it showed success in radiographic periapical healing (PAI < 3) (58.8% in the blood group, 94.1% in the S-PRF group, and 76.5% in the A-PRF group). There was no significant difference between the three groups in terms of the occurrence of healing scores at 6, 12, and 18 months (*p* = 0.442, *p* = 0.204, and *p* = 0.173), respectively.

Unfortunately, these methods have not been directly compared for periapical healing in adult mature tooth revascularization. However, for immature teeth, a meta-analysis conducted by Murray et al.,2018 evaluated the clinical efficacy of the revascularization in immature teeth after one year and concluded that the periapical lesion healing response is 88.9% for blood clot and 100% for PRF (*p* > 0.05) [39]. Although, *Sabeti *et al*., 2023* concluded that PRF exhibit the greatest success (75%) in periapical lesion resolution outcome within 12 months postoperatively compared to the blood clot technique (57%) [40].

To our knowledge for mature teeth studies, just one study comparing blood clot and standard PRF found that both groups' PAI scores of periapical lesions decreased significantly in the first 6 and 12 months after a REP. At 6 and 12 months, S-PRF showed a better periapical lesion healing rate than BC [11].

The repair of periapical lesions may have been affected by antimicrobial elimination and the early phase of the physiologic wound healing process, which is activated by tissue bleeding [41]. Additionally, blood contains immunoglobulins and cytokines from the innate and adaptive immune systems, which help locate and opsonize bacteria for phagocytosis [42].

All published research has shown that biological effects of autologous platelet concentrate depend on platelet concentration and number and type of leukocyte trapped in the fibrin membrane. S-PRF, a second-generation platelet concentrate, releases growth factors such as PDGF, TGF-β, b-FGF, and VEGF for up to ten days when used as a scaffold in REPs [43–45]. These growth factors control angiogenesis, promote stem cell migration, proliferation, and differentiation, and are essential for the self-regulation of infectious and inflammatory processes [17, 18]. Furthermore, S-PRF also forms a fibrin network that binds infection-fighting immune cells and cytokines and protects growth factors from proteolysis [46, 47].

A-PRF was treated like previous platelet concentrates, but with more evenly distributed platelets and leukocytes across the membrane, except for the acellular zone. Naturally, growth factors particularly IGF-1, EGF, and fibrin matrix protein impact A-PRF success. These factors have been found at higher concentrations than those found in blood and significantly enhance the process of tissue regeneration [48].

However, platelet rejuvenation is crucial because platelets can signal cell migration and start tissue healing. Masuki et al., [49] found that A-PRF didn't give good results regarding platelet renewal, which may explain its lower success rate (41.2%, 76.5%) compared to S-PRF (64.7%, 94.1%) at 12 and 18 months.

Most literature studies had limited follow-ups, which may underestimate failure rates. The current study has 18 months of follow-up, but long-term follow-up is needed to ensure long-term results. Even though 86.2% of teeth showed clinical success with the REPs, clinical failure after 18 months was 11.8% for both S-PRF and A-PRF and 17.6% for blood column.

According to the AAE, the ultimate objective of REPs should be a positive pulp vitality test which is commonly measured by electric or thermal test. Inaccurate results and considerable stimulus attenuation occur when cold testing is done because bioactive cements prevent cold stimuli from reaching living tissue [21]. It is critical to note positive pulp responses do not indicate canal space pulp tissue regeneration, and negative pulp responses do not indicate failure [10, 50]. Using regenerative endodontic therapy to restore pulp tissue's neuronal architecture is controversial and requires more research. Revitalization studies show increased sensitivity. In this regard, Arslan et al., [9] found that 50% of the teeth responded positively, agreeing with the findings of Nageh et al., [31] which found that 60% of the teeth responded, along with El-Kateb et al., [10] which also demonstrated the increased sensitivity of the treated teeth (77%).

*In the current study*, incidence of the regaining sensitivity after 12 months was 29.4% with A-PRF group and 41.2% within the S-PRF group, 17.6% with BC group. This is in line with the findings of Youssef et al., who observed that 20% of the BC group and 50% of the S-PRF group had recovered their pulp sensitivity [11]. The overall rate of regaining sensitivity with an 18-month follow-up was 29.4%, which is comparable to the findings of Lu et al., where 35% of teeth regained sensitivity after 4.3 years follow up [12].

Numerous reasonable avenues for regaining sensitivity exist, even if the specific procedure responsible for that after REPs is yet unidentified. An increase in the percentage of patients who regained tooth sensibility in the PRF group, compared to the BC group, may be attributed to firstly the different growth factors available in PRF, which is important for the neurogenesis process. Transforming growth factor beta regulates mitogenic effects of other growth factors (nerve growth factor and brain-derived neurotrophic factor), stimulates neurite outgrowth, and regulates differentiation of Schwann cells (SCs) [51]. Platelet derived growth factor (PDGF) induces SC proliferation, differentiation, and myelin formation [52]. Vascular endothelial growth factor promotes blood vessel growth, neurogenesis, and neuroprotection [53]. Insulin growth factor 1 supports the forward extension of the nerve fibers and suppresses apoptosis in motor, sensory, and sympathetic neurons [54]. Basic fibroblast growth factor facilitates neuroprotection and SC regeneration [55].

The ability of some dental pulp stem cells to survive a peri-radicular lesion and proliferate into neural cells, which initiates axon guidance, is the basis for the second theory [56]. According to the third mechanism, stem cells have the capacity to differentiate into neurogenic lineages and may originate from the periodontal ligament, which expresses markers of undifferentiated neural crest cells [57]. The bone marrow mesenchymal stem cells, which can develop into neurons and astrocytes under the right circumstances, are an integral component of the fourth potential mechanism [58]. The fifth potential technique involves the use of 17% EDTA irrigation, which stimulates neurogenesis by releasing neurotrophic factors from the dentin matrix locally. These elements are crucial for maintaining neurons because they encourage neuronal development and axonal regeneration [59]. The sixth proposed mechanism is that nearby ipsilateral nerves may have sprouted or ingrown, resulting in a collateral reinnervation of the pulp canal. Still, more research is necessary to understand the underlying mechanism of its activity [60].

Although this study's results seem encouraging, there are several constraints. The study's limitations include the inability to generalize findings across tooth kinds, a small number of participants, and a short follow-up period. However, future therapeutic directions will include extended monitoring and follow-up. It was difficult to induce blood in teeth with closed apices was another drawback. Another drawback was performance bias, which is unavoidable given the nature of the intervention. Finally, because the histological assessment of regenerated tissues is intrusive and necessitates tooth extraction, the findings cannot be correlated with them as well restoring pulp tissue after regenerative endodontic treatment is uncertain and requires clear data and objective testing.

Regeneration therapy is an attractive new alternative to traditional endodontic treatments. However, there are no established guidelines regarding REPs in mature teeth also, the prognosis of it is still vague, so a greater number of strong clinical investigations with prolonged follow up periods are needed. The higher success rates observed for PRF scaffolds could suggest a preference for PRF-based treatments in similar cases, which could be beneficial in guiding clinical decision-making.

Taking into consideration from the clinical point of view, before undergoing REPs, the patient must be well informed of the treatment possibilities and potential complications. In addition, the therapy might require longer follow-ups and stronger patient motivation. Furthermore, some endodontists may lack the training and experience to provide regenerative endodontic procedures [61]. Moreover, for future directions in REPs, it must be accompanied with respect to the following: new scaffolds, antimicrobial medicaments, the determination of pulpal blood flow might be a much better objective way to assess pulp vitality, also determentaion of potential markers like IL-8 or matrix metalloproteases MMP.

## Conclusion

The findings of this trial indicated that PRF based regenerative technique may outperform the blood clot technique in treatment of non-vital single mature teeth with chronic periapical periodontitis. There is a need for future randomized clinical studies to consolidate procedures in this field with more prolonged evaluation periods.

## Data Availability

The author confirms that all data generated or analysed during this study are included in this article.

## Abbreviations

REPs: Regenerative endodontic procedures
AAE: American Association of Endodontics
REC: Research Ethics Committe
BC: Blood column
APCs: Autologous platelet concentrates
S-PRF: Standard Platelet rich fibrin
A-PRF: Advanced platelet rich fibrin
PAI: Periapical index
DAP: Double antibiotic paste
TAP: Trible antibiotic paste
EDTA: Ethylenediaminetetraacetic acid
CEJ: Cemento-Enamel junction
CONSORT: Consolidated Standards of Reporting Trials
CBCT: Cone Beam Computed Tomography
